# Home Efficacy of a Postbiotic-Based Gel Compared with a Gel Without Active Ingredients for the Treatment of Gingival Inflammation in Patients with Down Syndrome: A Randomized Controlled Study

**DOI:** 10.3390/dj13020062

**Published:** 2025-01-30

**Authors:** Andrea Scribante, Paolo Appendino, Carolina Maiorani, Paolo Fontanarosa, Maurizio Pascadopoli, Sara Cammisuli, Basmala Azouz, Simone Buttiglieri, Andrea Butera

**Affiliations:** 1 Unit of Orthodontics and Pediatric Dentistry, Section of Dentistry, Department of Clinical, Surgical, Diagnostic and Pediatric Sciences, University of Pavia, 27100 Pavia, Italy; 2 Unit of Dental Hygiene, Section of Dentistry, Department of Clinical, Surgical, Diagnostic and Pediatric Sciences, University of Pavia, 27100 Pavia, Italy; 3 Department of Dentistry and Oral Surgery, Mauriziano Hospital, 10128 Turin, Italy

**Keywords:** periodontal disease, postbiotics, Down syndrome, adjunctive periodontal treatment

## Abstract

Objectives: The aim of this six-month randomized controlled study was to evaluate the efficacy of a non-surgical periodontal treatment combined with the use of an active gel compared to a non-surgical treatment alone in reducing inflammatory indices in periodontal patients with Down syndrome. Methods: A total of 40 patients were included in the study, 20 of which were assigned to the active group and 20 to the control group. The active group underwent non-surgical periodontal treatment supplemented by daily home application of an intensive soothing gel containing probiotics. The control group received non-surgical periodontal treatment combined with the application of a gel without active ingredients. The following clinical indices were assessed: Bleeding on Probing (BOP %), Plaque Control Record (PCR %), Mobility (Miller Index), and Modified Marginal Gingival Index (MGI). Measurements were taken at baseline (T0), one month after treatment initiation (T1), after three months (T2), and after six months (T3). The patient compliance was evaluated at each visit, and product satisfaction was assessed through a questionnaire using a Visual Analogue Scale (VAS). Results: By the end of the study, statistically significant improvements were observed in both the groups from T0 to T3 evaluation (*p* < 0.05). The BOP score was significantly lower in the Trial group at the T3 intergroup evaluation (*p* < 0.05). Conclusions: A soothing gel with postbiotic and natural compounds was a valuable adjunct to non-surgical periodontal treatment to improve periodontal health in patients with Down syndrome, reducing BOP after 6 months of treatment.

## 1. Introduction

According to the World Health Organization (WHO), the global incidence of Down syndrome (DS) is between 1 in 1000 and 1 in 1100 live births [1]. In Italy, the incidence is about 1 in 1200 live births, with an estimated 500 new cases per year and a total prevalence of about 38,000 affected individuals [2].

Individuals with DS are predisposed to a wide range of medical conditions, including neuropsychiatric disorders such as early onset dementia and autism spectrum disorders, congenital gastrointestinal anomalies, endocrine disorders such as hypothyroidism and diabetes mellitus, autoimmune diseases, congenital heart defects, obesity, osteoporosis, celiac disease, sleep apnea, and childhood leukemia [3].

Distinctive phenotypic features and orofacial anomalies are also frequently observed, including generalized muscle hypotonia; joint laxity; brachycephaly; a rounded face; upslanted palpebral fissures; dental anomalies affecting number, shape, and development; a high-arched palate; macroglossia with lip incompetence; and geographical and fissured tongue [4,5]. Anatomical, immunological, and physiological differences, combined with a reduced ability to manage the bacterial biofilm, increase the susceptibility of individuals with DS to periodontal disease, with a generally worse prognosis than non-DS individuals [6,7].

The progression of periodontitis in DS patients is linked to defects in innate immune responses, including reduced neutrophil chemotaxis and phagocytosis, and possibly a shortened neutrophil half-life [8]. These immune deficiencies impair the body’s natural defense mechanisms, limiting its ability to effectively counteract bacterial biofilm and contributing to the rapid progression of periodontal tissue destruction. Moreover, the altered inflammatory response seen in individuals with Down syndrome often leads to a chronic state of low-grade systemic inflammation, which may further exacerbate periodontal tissue breakdown and delay healing processes following treatment [8,9]. In addition to these biological challenges, environmental and social determinants also play a crucial role. Institutionalization, for example, has been associated with a negative impact on oral health, and particularly on the number of remaining teeth in individuals with Down syndrome. This can be attributed to multiple factors, including reduced access to routine dental care, insufficient resources for maintaining oral hygiene, and the lack of individualized care plans tailored to the specific needs of these patients. Furthermore, institutionalized settings may not always prioritize preventive dental interventions, leading to the increased accumulation of dental plaque, higher rates of periodontal disease progression, and ultimately, tooth loss [10]. These findings highlight the necessity of targeted oral health programs and comprehensive care strategies to address the unique challenges faced by institutionalized individuals with Down syndrome.

Additional factors negatively influencing periodontal health in DS patients include limited manual dexterity, a reduced understanding of oral hygiene procedures, and limited access to dental care [6,11]. Consequently, the bacterial plaque levels are generally high, while the prevalence of dental caries is paradoxically lower than in the general population [12,13]. The management of oral hygiene in these patients heavily relies on family members and caregivers, who play a crucial role in ensuring adequate biofilm control [14].

Standard therapeutic protocols include daily plaque control through assisted brushing, scaling, and root planing, with or without the use of local or systemic antibiotics or chlorhexidine [15]. However, conventional therapeutic approaches often yield unsatisfactory results in DS patients, underscoring the need for more tailored strategies [16,17]. However, it is crucial to consider that the prolonged use of chlorhexidine, while effective in controlling dental plaque and reducing gingival inflammation, must be carefully evaluated. Its extended application can lead to dysbiosis of the oral microbiome by selectively eliminating certain bacterial species and promoting the proliferation of others that may be potentially pathogenic [18]. Moreover, the use of antibiotics carries the risk of bacterial resistance and alterations in the oral microbiota [19]. Antibiotic-induced microbiota changes can persist for months or even years, compromising immune homeostasis and increasing the risk of recurrent infections [20]. In light of these challenges, there is growing interest in alternative solutions that can modulate the oral microbiota safely and effectively, such as prebiotics, probiotics, and postbiotics [21].

Postbiotics, unlike probiotics, are non-viable microbial products or metabolic byproducts of probiotic microorganisms. They are derived from the bioactive compounds that probiotics produce during their metabolism, including enzymes, peptides, polysaccharides, short-chain fatty acids, cell wall fragments, and other metabolites. These bioactive substances exert beneficial effects on the host’s health through mechanisms such as modulating the immune response, enhancing gut barrier function, and exerting anti-inflammatory or antioxidant properties [22,23].

A key advantage of postbiotics is that they provide similar clinical benefits to probiotics without containing live microorganisms, thereby eliminating the risks associated with administering live bacteria, especially in immunocompromised or critically ill individuals. Unlike probiotics, postbiotics do not carry the potential for infections, such as sepsis, or the risk of bacterial translocation, which has been a concern in high-risk populations [22]. While the clinical efficacy of probiotics is well documented in numerous studies, including their role in restoring the gut microbiota balance, preventing gastrointestinal infections, and modulating immune function, the occurrence of adverse events in vulnerable patients has prompted interest in safer alternatives. Postbiotics present a promising solution, offering the therapeutic benefits of probiotics while mitigating the associated risks, thus broadening their potential application in clinical and healthcare settings [23,24].

Postbiotics, therefore, represent a promising alternative, with immunomodulatory and anti-inflammatory effects that could improve the management of periodontitis in DS patients [25,26].

Due to the limited availability of scientific studies investigating the efficacy of postbiotics in the treatment of periodontitis in this population, the present randomized controlled study aims to evaluate the efficacy of a postbiotic-based gel compared to a placebo gel. The first null hypothesis of the study was that there were no significant intergroup differences regarding Bleeding on Probing (BOP) primary outcomes. The second null hypothesis was that there were no significant intergroup differences for all the secondary outcomes of the study.

## 2. Materials and Methods

### 2.1. Study Design

This study was a randomized, placebo-controlled trial, authorized by the Intercompany Ethics Committee of A.O.U. Città della Salute e della Scienza di Torino—A.O. Ordine Mauriziano—A.S.L. “Città di Torino” (CE 564/2022). The trial protocol has been registered on clinicaltrials.gov (NCT: NCT06293911).

### 2.2. Participants

The study population consisted of patients with Down syndrome. The trial was conducted at the Odontostomatology Unit of the Azienda Ospedaliera Ordine Mauriziano of Turin. Inclusion criteria were as follows: patients with Down syndrome, non-bedridden (including edentulous patients), aged between 18 and 70 years, presenting with gingival inflammation characterized by marginal edema, erythema, Bleeding on Probing (BoP), and clinical signs of inflammation, as defined by the 2017 World Workshop classification of periodontal diseases. Informed written consent was obtained from the participants or their legal guardians before enrollment in the study. Exclusion criteria were as follows: patients with cardiac pacemakers, individuals undergoing oncological treatment, those who had received bisphosphonates within the last 12 months, and patients with lifestyle factors incompatible with the study protocol (e.g., substance abuse or alcohol dependency).

### 2.3. Intervention and Outcomes

At baseline (T0), after obtaining informed consent, patients underwent clinical periodontal examination. The following clinical indices were assessed by a calibrated operator using a UNC 15 periodontal probe (Hu-Friedy, Chicago, IL, USA): BoP, Bleeding on Probing (percentage of sites exhibiting bleeding following periodontal probing) [27]; PCR, Plaque Control Record (percentage of tooth surfaces with visible plaque accumulation) [28]; MGI, Modified Gingival Index (qualitative assessment of gingival inflammation based on visual inspection) [29]; Mobility (assessed with Miller Index) [30].

All patients received initial non-surgical periodontal therapy, consisting of supra- and subgingival debridement using a periopolishing system with glycine powder to reduce microbial biofilm and eliminate etiological factors (Combi Touch, Mectron S.p.A., Carasco, GE, Italy). Following this, participants were randomly assigned to one of the two treatment groups: Group 1 (Trial group) applied a gingival gel with postbiotics once daily on gingival tissues until the next scheduled follow-up; Group 2 (control group) applied a placebo gel with no active ingredients, following the same protocol (placebo gel, Coswell S.p.A., Funo di Argelato, BO, Italy) (Table 1).

The two gels under examination have similar compositions but differ in some active ingredients that may influence their effectiveness, like Xylitol, Zinc Hydroxyapatite, Zinc PCA, Aloe Barbadensis Leaf Juice Powder, Lactobacillus Ferment, Sodium Hyaluronate, Lactoferrin, and Solidago Virgaurea Extract.

Both formulations contain Xanthan Gum and Silica, which act as stabilizers and mild abrasive agents. However, the second gel includes active ingredients such as Zinc Hydroxyapatite and Lactoferrin, which help strengthen tooth enamel and fight pathogenic bacteria. Sodium Hyaluronate and Aloe Barbadensis Leaf Juice Powder are present in both gels and are known for their hydrating and soothing properties. Additionally, the second gel also contains Lactobacillus Ferment, which promotes an environment favorable to oral microbiota.

These differences in active components can lead to variations in the effectiveness of the gels, but also in their potential side effects. For instance, ingredients such as Aroma, Linalool, and Limonene present in both formulations may cause irritation or allergic reactions in sensitive individuals [31], while preservatives like Phenoxyethanol and Sodium Benzoate could cause discomfort to the oral mucosa [32].

The first follow-up visit (T1) was conducted one month after the initial treatment, during which periodontal indices were reassessed, and additional periopolishing with glycine powder was performed on any persistently inflamed sites. Subsequent evaluations were conducted at 3 months (T2) and 6 months (T3) post-treatment. At each follow-up, the same clinical parameters were recorded, while professional oral hygiene treatment was repeated only at the T2 and T3 visits.

Additionally, patient compliance was assessed through a Visual Analogue Scale (VAS), focusing on adherence to the prescribed regimen, any lifestyle modifications, and punctuality in attending scheduled follow-up: scores from 0 to 2 indicated poor compliance, scores from 3 to 5 indicated sufficient compliance, scores from 6 to 8 indicated good compliance, and scores from 9 to 10 indicated excellent compliance. Product satisfaction was evaluated using the same scale, analyzing attributes such as taste, odor, texture, persistence, and ease of application. The evaluation scale assigned the following ratings: 0 for insufficient, 1 to 3 for sufficient, 4 to 6 for good, 7 to 9 for very good, and 10 for excellent.

### 2.4. Randomization

A data analyst used a block randomization table to generate a sequence, applying a permuted block of 40 patients in accordance with the study design. The Trial treatment was randomly assigned to one patient, while the subsequent patient was assigned to the Control treatment. To maintain allocation concealment, opaque, sealed envelopes were prepared in advance and sequentially numbered (SNOSE). Subsequently, a designated operator performed the procedures and collected the required index data. To maintain blinding, both the patients and the data analyst were blinded to the treatment allocation, and the two gels for home oral hygiene were appropriately masked. The gels were differentiated by distinct colors, and written instructions were provided on the packaging to minimize errors and ensure adherence to the split-mouth protocol.

### 2.5. Sample Size

The sample size was calculated with an alpha of 0.05 and a power of 85% for two independent study groups. The primary continuous endpoint, Bleeding on Probing (primary outcome), was hypothesized to have a mean of 41, with an expected mean difference of 19 and a standard deviation of 20 [33]. Based on these parameters, 20 patients per group were required for the study.

### 2.6. Statistical Analysis

Data analysis was performed at the Experimental Testing Laboratory of the Unit of Orthodontics and Pediatric Dentistry, Department of Clinical-Surgical, Diagnostic, and Pediatric Sciences, University of Pavia. Statistical evaluation was performed using R software (R version 3.1.3, R Development Core Team, R Foundation for Statistical Computing, Vienna, Austria). Descriptive statistics were calculated for each variable, including mean, standard deviation, median, and minimum and maximum values for both treatment groups.

The normality of data distribution was assessed using the Kolmogorov–Smirnov test. Subsequently, the Friedman test followed by Dunn’s post hoc test for multiple comparisons were performed. Statistical significance was set at *p* < 0.05 for all the tests.

## 3. Results

### 3.1. Baseline Demographic Data and Flow Chart Participants

**Figure 1:**
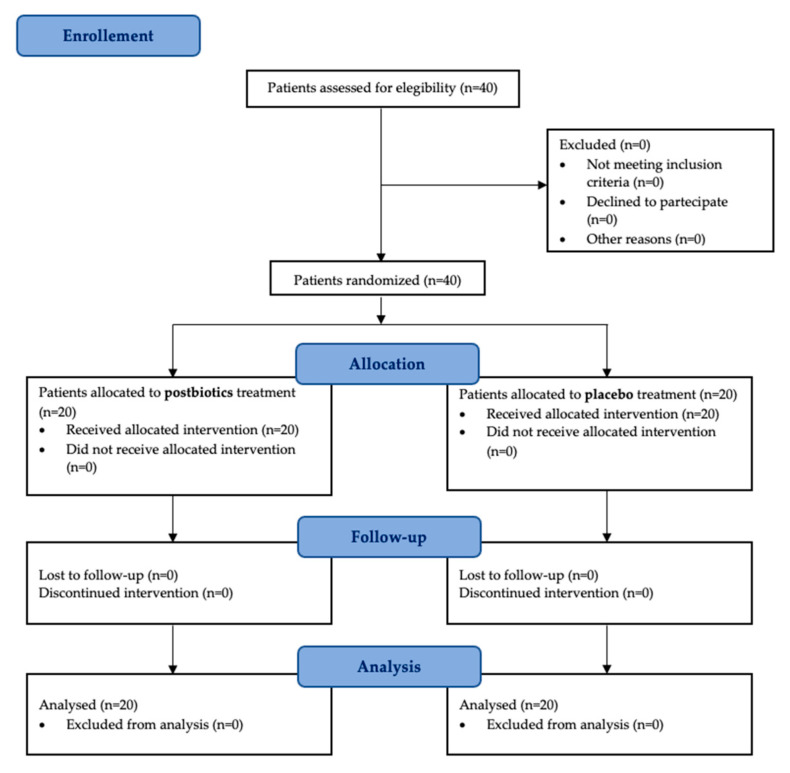
CONSORT flow chart of the study showing enrollment and allocation procedures.

A total of 40 patients (22 males and 18 females) met the inclusion criteria, provided informed consent, and received the assigned interventions. The study started in March 2024 and ended in December 2024. All of the enrolled patients were included in the final analysis without any exclusions (Figure 1). Their demographic baseline data are shown in Table 2.

The results of the multiple comparisons from the inferential statistics are presented using a letter-based presentation, in which means presenting the same letters do not present significant differences [34].

### 3.2. Bleeding on Probing

The Bleeding on Probing in the Trial group significantly improved from T0 to both T2 and T3 (*p* < 0.05), while no significant change was noted between T0 and T1 (*p* > 0.05). In contrast, the control group showed no significant changes in BOP during any of the follow-up visits (*p* > 0.05). A significant difference between the groups was observed at T3 (*p* < 0.05) (Table 3).

### 3.3. Plaque Control Record

The mean Plaque Control Record (PCR) in the Trial group significantly decreased from between the T0 and T2 (*p* < 0.05) to T0 and T3 (*p* < 0.05) comparisons, whereas no statistically significant difference was detected between T0 and T1 (*p* > 0.05). In the control group, no significant changes in the PCR were observed across the various follow-ups. Likewise, no statistically significant differences between the groups were observed at any time point (Table 4).

### 3.4. Modified Gingival Index

The mean Modified Gingival Index (MGI) score in the Trial group underwent significant improvements at all follow-up visits (T1, T2, and T3) compared to baseline (*p* < 0.05). In contrast, the control group showed no significant changes in the MGI across the follow-up periods (*p* > 0.05), and no significant intergroup differences were found (*p* > 0.05) Table 5.

### 3.5. Miller Mobility

In the Miller Mobility Index score, instead, no significant intergroup or intragroup differences were found at any time frames (*p* > 0.05) (Table 6).

### 3.6. Patient Compliance

The mean Visual Analogue Scale (VAS) score for patient compliance resulted in a significant improvement from T0 to T3 in the Trial group (*p* < 0.05), whereas no significant changes were recorded in the control group throughout the follow-up visits (*p* > 0.05) (Table 7).

### 3.7. Patient Satisfaction

**Figure 2:**
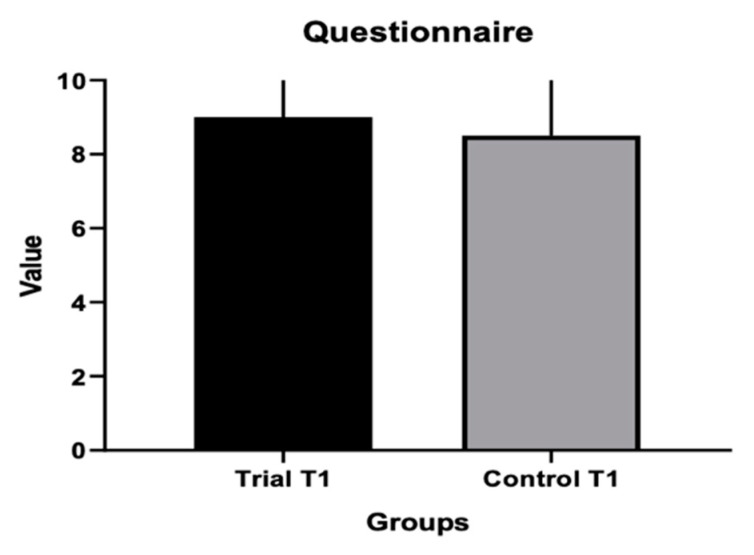
Satisfaction scale scores.

Regarding the score for the satisfaction with the products, no significant difference between the two groups was found (*p* > 0.05) (Figure 2).

### 3.8. Harms

No harm or unintended effects were reported in the two study groups.

## 4. Discussion

DS is associated with numerous health issues, including intellectual disabilities and various medical conditions such as heart diseases, gastrointestinal disorders, diabetes, obesity, and endocrine abnormalities [35]. Among these, an increased predisposition to developing periodontal diseases has been observed [5], a condition exacerbated by anatomical and systemic factors such as compromised immune response, as well as behavioral factors [9]. Additionally, other conditions like malocclusions and obstructive sleep apnea have been linked to alterations in the host’s oral microbiome eubiosis [36].

The standard treatment for periodontitis in patients with DS is scaling and root planing (SRP). Several studies have documented the effectiveness of this therapy when combined with chlorhexidine, administered as a mouthwash or gel in various concentrations [15,37,38].

Other studies have investigated the use of antibiotics, such as amoxicillin and metronidazole, as adjuncts to subgingival debridement, highlighting their efficacy in reducing inflammatory indices and periodontal pocket depths [39,40,41]. Nevertheless, further studies are required to consolidate these findings.

The prolonged use of chlorhexidine and antibiotics, particularly in high-risk patients, must be carefully evaluated as it may lead to dysbiosis, the proliferation of potentially pathogenic bacterial species, and antimicrobial resistance [18]. As a result, these therapies are less suitable for patients with DS, in whom periodontitis is characterized by a cycle of compensation and amplification of the inflammatory response, leading to chronic and persistent inflammation. Therefore, a therapeutic approach that promotes long-term periodontal health in these patients is essential.

An alternative approach to modulating the oral microbiota in periodontal patients involves the use of probiotics. A systematic review on the efficacy of probiotics as an adjunct to non-surgical periodontal therapy revealed a positive clinical effect on several parameters, including the probing pocket depth (PPD), clinical attachment level (CAL), Bleeding on Probing (BoP), and plaque index (PI), in the short term (up to 3 months). However, the wash-out effect of probiotics observed after 6 months limits their long-term efficacy [42].

Periodontitis is a chronic inflammatory condition that leads to the progressive destruction of the tissues supporting teeth, often associated with microbial imbalances in the oral cavity. Treatments aim to restore microbial harmony by reducing the population of key pathogens such as *Aggregatibacter actinomycetemcomitans* (Aa), a bacterium commonly linked to aggressive forms of the disease. Given the challenges posed by resistance to traditional antimicrobials, alternative strategies involving beneficial microbes and their byproducts, known as postbiotics, have gained traction as innovative approaches to managing periodontal disease [43].

Preliminary investigations have demonstrated that cell-free supernatants (CFSs) derived from specific Lactobacillus strains, including *L. rhamnosus Lr32*, *L. rhamnosus HN001*, *L. acidophilus LA5*, and *L. acidophilus NCFM*, can suppress Aa biofilm formation and partially reduce the number of viable bacteria. These supernatants also modulate the expression of virulence genes such as cdtB and ltxA, with LA5 and Lr32 showing particularly notable effects [43]. Similarly, postbiotic metabolites (PMs) derived from *Lactiplantibacillus plantarum PD1*8, selected from 139 isolates, exhibited strong inhibitory activity against pathogens like *Streptococcus mutans*, *Porphyromonas gingivalis*, *Tannerella forsythia*, and *Prevotella loescheii*. The PD18 PM achieved significant biofilm reduction—92.95% for S. mutans and 89.68% for *P. gingivalis*—with minimal inhibitory concentrations (MICs) ranging from 1:2 to 1:4. These results suggest that postbiotic compounds could serve as effective natural adjuncts for preventing biofilm-associated infections [44].

In previous research, 30 patients undergoing scaling and root planing were divided into two groups for home treatment: one group used a gel containing postbiotic compounds, while the other used a chlorhexidine-based gel. Both treatments significantly improved clinical parameters such as the probing pocket depth, Bleeding on Probing, and Plaque Control Record over six months, though no major differences emerged between the groups. These findings reinforce the potential of postbiotics in reducing pathogen virulence, controlling inflammation, and supporting periodontal health. However, further studies are needed to refine their use and identify the precise mechanisms driving their therapeutic effects [45].

The use of postbiotics in this research was motivated by the need to modulate the oral microbiome safely and effectively, particularly in patients with DS, who are at higher risk for complications. Postbiotics, derived from microbial metabolites produced during fermentation or cell lysis, do not contain live microorganisms and offer benefits to the host without the risks associated with probiotics, such as bacteremia or fungemia [26]. Furthermore, through mechanisms such as the production of organic acids and bacteriocins, postbiotics exhibit significant antimicrobial activity, creating an environment unfavorable for pathogenic bacterial growth [46]. These properties make them a promising adjunct in periodontal therapy for managing gingival inflammation in patients with DS. Indeed, this clinical study clearly demonstrates that the use of a gel containing postbiotics is effective in significantly reducing Bleeding on Probing in patients undergoing non-surgical periodontal treatment. This positive effect can be attributed to the beneficial action of postbiotics, which help improve the health of oral bacterial flora and modulate the inflammatory response in periodontal tissues [43,45]. The results suggest that the use of this formulation can provide valuable support in periodontal therapies, improving patient management and promoting more effective healing.

Moreover, high patient compliance was observed, a crucial and determining factor for the success of non-surgical periodontal therapy. The patient’s cooperation in following the professional’s instructions, including the regular application of the gel and the adoption of proper at-home oral hygiene practices, played a fundamental role in achieving the therapeutic goals. This finding underscores the importance of educating patients about the value of their active participation in treatment, a key element for maintaining the benefits achieved over the long term and preventing the recurrence of periodontal disease [47].

The first null hypothesis was rejected, as significantly lower BOP scores were found in the Trial group (*p* < 0.05) at the T3 evaluation.

The second null hypothesis, instead, was accepted, as no significant intergroup differences were found between the two groups in any time frame.

However, a tendency in both the groups to a significant intragroup decrease was found, highlighting the positive effect of non-surgical periodontal debridement.

An essential factor for treatment success is communication with the parents and legal guardians of patients. This requires a clear understanding of the recommended procedures and a commitment to integrating these practices into daily routines. However, long-term compliance is often hindered by motor and cognitive limitations, as well as the presence of early-onset dementia, which further compromises cooperation and adherence to the treatment plan. These challenges can be particularly pronounced in cases where additional support systems are lacking, creating a cycle that further complicates effective treatment adherence. Additionally, limited access to dental care represents a significant issue, with one study reporting that only 69.2% of dentists regularly treat patients with disabilities [9].

It is therefore imperative to develop personalized care plans that address the specific needs of each patient and promote an inclusive and empathetic approach. Regular visits and effective communication not only enhance therapeutic outcomes but also contribute to building a trust-based relationship between the healthcare professional and the patient, which is essential for maintaining long-term results.

The limitations of this study should be considered when interpreting the findings. Additionally, the study was conducted at a single center, which may restrict the applicability of the findings to other settings with different demographic or clinical characteristics. The follow-up duration, while sufficient to observe short-term changes, does not account for long-term outcomes or the sustainability of the intervention’s effects. Furthermore, the reliance on visual indices introduces potential observer bias, despite the calibration efforts for assessment consistency. Lastly, the compliance of the patients cannot be assessed at all for home treatment.

Future studies with extended follow-up periods are needed to validate these findings and explore the long-term efficacy and safety of postbiotic-based therapies in this population.

## 5. Conclusions

The results of this randomized, placebo-controlled study demonstrated that the daily application of an intensive soothing gel containing postbiotics and natural ingredients significantly improved the periodontal health of individuals with DS. Specifically, a notable reduction in the BOP index was observed after 6 months of consistent home treatment. These findings highlight the potential of incorporating such innovative formulations into routine oral care for individuals with DS, offering a promising strategy to address the unique periodontal challenges faced by this population. Furthermore, the use of natural ingredients and postbiotics underscores the growing importance of safe, effective, and accessible interventions in enhancing overall oral health.

## Figures and Tables

**Table 1:** Products tested in the study.

Gel	Manufacturer	Composition
Biorepair Plus Parodontgel Intensive	Coswell S.p.A., Funo di Argelato, BO, Italy	Aqua, Propylene Glycol, Peg-40 Hydrogenated Castor Oil, Xylitol, Xanthan Gum, Silica, Zinc Hydroxyapatite, Zinc PCA, Aloe Barbadensis Leaf Juice Powder, Lactobacillus Ferment, Sodium Hyaluronate, Lactoferrin, Solidago Virgaurea Extract, Aroma, Sodium Benzoate, Phenylpropanol, Benzyl Alcohol, Hydroxyacetophenone, Sodium Saccharin, O-Cymen-5-ol, Mannitol, Decylene, Glycol, Sodium Myristoyl Sarcosinate, Sodium Methyl Cocoyl Taurate, Citric Acid, Potassium Sorbate, Phenoxyethanol, Linalool, Benzyl Benzoate, Limonene.
Placebo gel	Coswell S.p.A., Funo di Argelato, BO, Italy	Aqua, Propylene Glycol, Peg-40 Hydrogenated Castor Oil, Xanthan Gum, Silica, Aroma, Sodium Benzoate, Phenylpropanol, Benzyl Alcohol, Hydroxyacetophenone, Sodium Saccharin, O-Cymen-5-ol, Mannitol, Decylene, Glycol, Sodium Myristoyl Sarcosinate, Sodium Methyl Cocoyl Taurate, Citric Acid, Potassium Sorbate, Phenoxyethanol, Linalool, Benzyl Benzoate, Limonene.

**Table 2:** Demographic data of the study sample.

Patients	Sex	n (%)	Mean Age (SD)
Total	Males	22 (55.00%)	31.73 (8.74)
	Females	18 (45.00%)	29.28 (9.17)
Trial	Males	11 (27.5%)	32.82 (7.41)
	Females	9 (22.5%)	30.11 (7.9)
Control	Males	11 (27.5%)	30.64 (10.14)
	Females	9 (22.5%)	29.28 (9.17)

Legend: n, number of patients; SD, standard deviation.

**Table 3:** BOP scores.

Group	Time	Mean	St Dev	Min	Median	Max	Significance *
Trial	T0	54.95	28.96	7.00	52.00	100.00	A
	T1	31.70	19.75	2.00	34.50	70.00	A, B
	T2	25.05	14.86	4.00	28.50	55.00	B, C
	T3	25.35	11.94	5.00	27.00	50.00	C
Control	T0	49.65	28.60	10.00	45.50	100.00	A
	T1	40.55	27.46	2.00	37.50	87.00	A, B
	T2	40.10	24.52	0.00	33.00	85.00	A, B
	T3	35.90	23.86	4.00	30.00	90.00	A, B

* Means with the same letter are not significantly different (*p* > 0.05).

**Table 4:** PCR scores.

Group	Time	Mean	St Dev	Min	Median	Max	Significance *
Trial	T0	79.00	22.93	35.00	87.50	100.00	A
	T1	60.05	17.69	22.00	63.00	100.00	A, B
	T2	58.65	19.56	20.00	62.50	87.00	B
	T3	56.65	23.82	20.00	60.00	100.00	B
Control	T0	79.65	18.54	50.00	80.50	100.00	A
	T1	69.75	23.84	30.00	65.00	100.00	A, B
	T2	69.5	22.64	32.00	61.00	100.00	A, B
	T3	66.00	26.24	12.00	61.50	100.00	A, B

* Means with the same letter are not significantly different (*p* > 0.05).

**Table 5:** MGI scores.

Group	Time	Mean	St Dev	Min	Median	Max	Significance *
Trial	T0	2.44	0.60	1.00	2.40	3.00	A
	T1	1.51	0.53	1.00	1.5	2.20	B, C
	T2	1.25	0.72	0.00	1.00	2.00	C
	T3	1.16	0.69	0.00	1.00	2.00	C
Control	T0	2.05	0.69	1.00	2.00	3.00	A, B
	T1	1.65	0.88	0.00	2.00	3.00	B, C
	T2	1.85	0.67	1.00	2.00	3.00	A, C
	T3	1.80	0.70	1.00	2.00	3.00	A, C

* Means with the same letter are not significantly different (*p* > 0.05).

**Table 6:** Mobility Index scores.

Group	Time	Mean	St Dev	Min	Median	Max	Significance *
Trial	T0	0.55	0.76	0.00	0.00	2.00	A
	T1	0.40	0.50	0.00	0.00	1.00	A
	T2	0.35	0.49	0.00	0.00	1.00	A
	T3	0.15	0.37	0.00	0.00	1.00	A
Control	T0	0.50	0.76	0.00	0.00	2.00	A
	T1	0.50	0.76	0.00	0.00	2.00	A
	T2	0.50	0.76	0.00	0.00	2.00	A
	T3	0.45	0.69	0.00	0.00	2.00	A

* Means with the same letter are not significantly different (*p* > 0.05).

**Table 7:** VAS scores.

Group	Time	Mean	St Dev	Min	Median	Max	Significance *
Trial	T0	5.75	2.83	2.00	5.00	10.00	A
	T1	4.55	2.35	2.00	4.00	10.00	A, B
	T2	4.00	2.20	1.00	4.00	8.00	A, B
	T3	3.60	2.11	0.00	3.50	8.00	B
Control	T0	5.75	3.16	0.00	5.50	10.00	A
	T1	5.35	2.89	0.00	5.50	10.00	A, B
	T2	5.40	2.96	0.00	6.00	10.00	A, B
	T3	5.40	2.85	0.00	5.50	10.00	A, B

* Means with the same letter are not significantly different (*p* > 0.05).

## Data Availability

Data are available upon reasonable request to the corresponding authors.
